# Hepatitis E virus ORF2 protein over-expressed by baculovirus in hepatoma cells, efficiently encapsidates and transmits the viral RNA to naïve cells

**DOI:** 10.1186/1743-422X-8-159

**Published:** 2011-04-08

**Authors:** Mohammad K Parvez, Robert H Purcell, Suzanne U Emerson

**Affiliations:** 1 Molecular Hepatitis Section, Laboratory of Infectious Diseases, National Institute of Allergy and Infectious Diseases, National Institutes of Health, 50 South Drive, Bethesda, MD 20892-8009, USA; 2 Hepatitis Viruses Section, Laboratory of Infectious Diseases, National Institute of Allergy and Infectious Diseases, National Institutes of Health, 50 South Drive, Bethesda, MD 20892-8009, USA; 3 Department of Pharmacognosy, King Saud University College of Pharmacy, Riyadh, KSA

## Abstract

A recombinant baculovirus(vBacORF2) that expressed the full-length ORF2 capsid protein of a genotype 1 strain of hepatitis E virus(HEV) was constructed. Transduction of S10-3 human hepatoma cells with this baculovirus led to large amounts of ORF2 protein production in ~50% of the cells as determined by immune fluorescence microscopy. The majority of the ORF2 protein detected by Western blot was 72 kDa, the size expected for the full-length protein. To determine if the exogenously-supplied ORF2 protein could transencapsidate viral genomes, S10-3 cell cultures that had been transfected the previous day with an HEV replicon of genotype 1 that contained the gene for green fluorescent protein(GFP), in place of that for ORF2 protein, were transduced with the vBacORF2 virus. Cell lysates were prepared 5 days later and tested for the ability to deliver the GFP gene to HepG2/C3A cells, another human hepatoma cell line. FACS analysis indicated that lysates from cell cultures receiving only the GFP replicon were incapable of introducing the replicon into the HepG2/C3A cells whereas ~2% of the HepG2/C3A cells that received lysate from cultures that had received both the replicon and the baculovirus produced GFP. Therefore, the baculovirus-expressed ORF2 protein was able to trans-encapsidate the viral replicon and form a particle that could infect naïve HepG2/C3A cells. This ex vivo RNA packaging system should be useful for studying many aspects of HEV molecular biology.

## Findings

Hepatitis E virus (HEV) causes acute hepatitis which has an overall fatality rate of about 2%[1,2]: however, in developing countries, hepatitis E mortality rates may approach 20% in pregnant women[3,4]. HEV, currently the only member of the family *Hepeviridae*, is classified into 4 genotypes. Genotypes 1 and 2 infect only humans and non-human primates whereas genotypes 3 and 4 are zoonotic and infect swine and some other mammals in addition to humans[5]. The HEV genome is a linear, single-stranded, positive sense RNA of ~7.2 kb. It contains 3 open reading frames (ORFs)[6,7]. ORF1 encodes a non-structural polyprotein essential for virus replication. ORF3 codes for a very small protein which has putative regulatory functions[8] and which is required for release of virus from infected cells[9]. ORF2 encodes the viral capsid protein; although the full-length capsid protein consists of 660 amino acids, the apparent susceptibility of ORF2 protein to proteolytic cleavage means the size of the protein in virions is not known. The size of ORF2 protein varies when over-expressed in insect or mammalian cells and ORF2 products of 52-84 kDa have been reported[10-13]. A truncated ORF2 protein(53 kDa) expressed in insect cells was shown to assemble into empty capsids with a T1 symmetry[11] and an almost full-length ORF2 protein produced in insect cells was found to assemble into virus particles with T3 symmetry; these particles captured some of the ORF2-encoding mRNA[14]. However, trans-encapsidation of infectious virion RNA by over-expressed recombinant ORF2 protein has not been reported. HEV has been difficult to grow in cell culture and although recent advances in the culturing of genotypes 3 and 4 have occurred[15,16], each virus isolate requires adaptation by lengthy passage in cell culture to grow efficiently. Additionally, comparable systems for genotypes 1 and 2 have yet to be developed. Therefore, it would be useful to have a means of producing infectious virions of HEV by a process that did not require lengthy adaptation to cell culture

**Figure 1:**
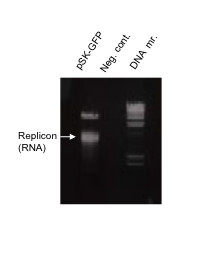
***In vitro *transcription of HEV genomic replicon**. The *Bgl*II-linearized plasmid, pSK-GFP(replicon), (5 μg) was transcribed in a 50 μl reaction volume and cooled on ice. A 2.5 μl of the RNA mixture was checked for RNA integrity and semi-quantitation on a 1% agarose gel, containing ethidium bromide. DNA. mr.(standard DNA marker); Neg.cont.(negative control).

**Figure 2:**
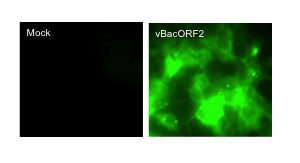
**Expression of HEV ORF2 in vBacORF2-transduced S10-3 cells**. Immune-staining(primary anti-ORF2 and secondary Alexa Fluor 488 goat anti-human IgG) of S10-3 cells at day 5 post-transduction, showing over-expression of ORF2 protein.

In the present report, we investigated whether recombinant baculovirus-mediated trans-complementation with HEV capsid protein could lead to packaging of an HEV replicon and its subsequent transmission to naive hepatoma cells. ORF2 and ORF3 are translated from a bicistronic, subgenomic mRNA. Therefore, production of either protein can be used as an indirect indicator of viral replication. In the present case, the ORF2 coding sequence had been substituted with that of green fluorescent protein (GFP) and GFP production was used to monitor viral replication[17]. Cultures of human S10-3 and HepG2/C3A cells[18,19] and insect Sf9 cells (Novagen)[20] were maintained as previously described. S10-3 cells were seeded (0.5 × 10^6 ^cells/well, in triplicate), in a 12-well culture plate. The Bgl*II*-linearized pSK-GFP (SAR55 HEV replicon) was transcribed *in vitro*(Figure 1) and transfected into S10-3 cells, essentially as described elsewhere[18]. Cells were incubated at 34.5°C with 5% CO_2 _and observed by fluorescence microscopy (FM)(Zeiss) for GFP production. Approximately 50% of the cells contained detectable GFP at day 6(data not shown). A recombinant baculovirus over-expressing ORF2 protein was constructed by inserting SAR55 HEV sequences encoding ORF3/ORF2, nt. 5130-7204 into the pTriEx1.1 vector (Novagen) at the Nco*I *and Bgl*II *sites, in frame with the vector start codon. Expression of ORF2 from the final transfer vector, pTriEx-ORF2 (~7.3 kb) was further directed by deleting the upstream ORF3 start codon sequences by polymerase chain reaction-based site-directed mutagenesis(TaKaRa Bio Inc). Plaques of recombinant baculo-ORF2 virus (vBacORF2) were isolated from Sf9 monolayers according to the manufacturer's instructions (BacVector kit, Novagen). vBacORF2 were amplified and a virus stock (10^7 ^pfu/μl) was prepared by pelleting the virus through a 5% and onto a 40% sucrose cushion (prepared in 1× PBS) in a SW28 Beckman rotor at 26,000 rpm [20]. S10-3 cells were transduced at a multiplicity of infection (moi) of 100, as described previously[20], except that OptiMem(Invitrogen) was substituted for serum-free DMEM. The next day, S10-3 cells were re-seeded in duplicate in 8-chamber glass slides in complete medium and incubated at 37°C. Cells were immune-stained on days 2 and 5 with primary anti-ORF2 (chimp1313-sera) and secondary antibody-conjugate (Alexa Fluor 488 goat anti-human IgG, Molecular Probes) as described previously[18]. The slides were mounted (VECTASHIELD HardSet Mounting Medium with DAPI, Vector Laboratories) and observed with the 25× objective of an indirect fluorescence microscope(IFM)(Zeiss) and a FITC filter. A very high level of HEV capsid protein was detected in ~50% of transduced cells (Figure 2).

**Figure 3:**
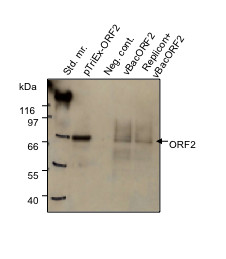
***In vitro *and *ex vivo *expression of full length ORF2 protein**. WB analysis, showing *in vitro *(TNT coupled transcription-translation of pTriEx-ORF2) as well as ex *vivo *(day 2, post-transduction with vBacORF2) translations of full length ORF2 (~72 kDa band). Std. mr.(standard protein marker); Neg.cont.(negative control).

Western blot (WB) analysis was performed at days 2 and 5 post-transduction to confirm the molecular mass of the *ex vivo *expressed ORF2 protein and to validate its integrity. Briefly, pelleted S10-3 cells from one well of a 12-well plate were lysed by vortexing in 90 μl water, then 10 μl of 10× PBS plus 7.1 μl of a 25% solution of NP40 were added and the lysate was incubated for 10 min at room temperature. The lysates were cleared at 15,600 × g and stored at -20°C. Additionally, the pTriEx-ORF2 vector was transcribed and translated *in vitro *in a 50 μl reaction volume (TNT-Coupled Reticulocyte Lysate System, Promega) as per manufacturer's instructions and stored at -20°C. Twenty four μl of each sample (*in vitro *and *ex vivo *preparations), was denatured in NuPAGE SDS buffer and reducing agent(Invitrogen). The protein samples were subjected to electrophoresis on a NuPAGE 7% Tris-acetate polyacrylamide gel (Invitrogen) followed by transfer onto a nitrocellulose membrane(Invitrogen). Blocking and antibody detection steps were performed, using the Snap i.d. protein detection system (Millipore). The membrane was washed in StartingBlock-TBS (Millipore) plus 1% Tween-20(Pierce), and incubated with chimp1313 anti-ORF2 sera for 10 min at RT followed by overnight incubation at 4°C. The blot was washed and further incubated with AffiniPure peroxidase-conjugated rabbit anti-human IgG(Jackson Immuno Research) for 10 min at RT, followed by treatment with SuperSignal West Femto Maximum Sensitivity Substrate(Thermo Scientific). The blot was subsequently exposed to an X-ray film (Kodak) and developed. The WB of *in vitro *translated ORF2 showed a ~72 kDa product that co-migrated with the *ex vivo *product expressed at day 2 (Figure 3). Lysate from day 5 post-transduction also contained a smaller band of ~55 kDa that was assumed to represent a processed form of 72 kDa protein (data not shown), in line with earlier reports [10-13].

**Figure 4:**
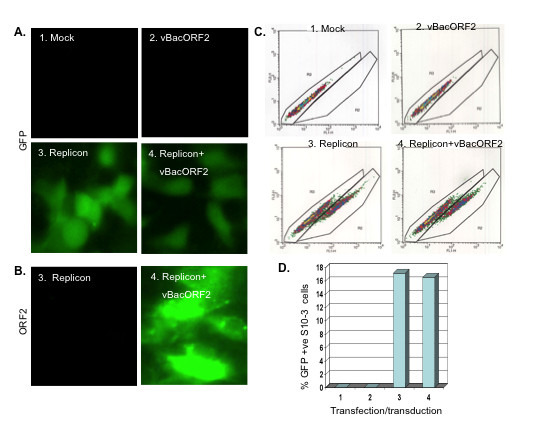
**Co-expression of ORF2 and GFP proteins in HEV replicon-transfected cells in a culture**. (A) FM, showing GFP expression in cells at day 6 post-transfection. (B) IFM, showing ORF2 expression in cells at day 5 post-transduction. (C) FACS plot, showing expression of GFP in cells at day 6. (D) %GFP positive cells in C, determined by FACS.

Controls were performed to demonstrate that baculovirus-expressed ORF2 protein and replicon-expressed GFP could be detected in the same culture. S10-3 cells were transduced one day after transfection with the replicon. As observed by fluorescence microscopy at day 6, the replicon-transfected cells expressed GFP while the mock-transfected and vBacORF-transduced cells were negative for green fluorescence (Figure 4A). Cell cultures that received both the replicon and vBacORF2 or vBacORF2 alone, were immunostained for ORF2 protein and an estimated 50% cells were positive on day 6 (Figure 4B). Note that the acetone fixation step in the IFM procedure destroyed the GFP signal. FACS analysis for GFP expression demonstrated that the assay was specific for GFP and that baculovirus transduction did not change the number of cells expressing GFP. S10-3 cells in a 24-well plate were harvested at day 5 post-transduction by treatment with 100 μl trypsin (Invitrogen) per well followed by 200 μl of 1 × PBS. Wells were rinsed with 200 μl more PBS and liquids were pooled (~500 μl/tube, final). The cells were pelleted at 4°C, and re-suspended in 300 μl of cold PBS on ice. The samples were immediately subjected to flow cytometry and a total of 10,000 cells were counted for every sample (Figure 4C). The FACS analysis demonstrated that about 16% of the cells were GFP positive whether or not the cells were transduced (Figure 4D).

**Figure 5:**
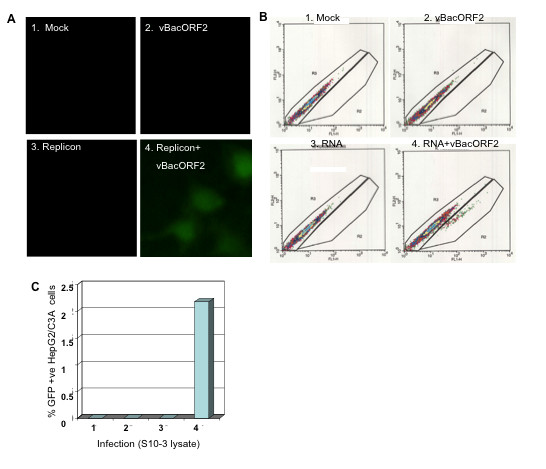
**Infectivity assay with naïve HepG2 cells**. (A) FM, showing expression of GFP in virion (S10-3 lysate)-infected cells (~5%), at day 6. (B) FACS plot, showing GFP expressing cells at day 6 post-infection. (C) %GFP positive cells in B, determined by FACS.

In order to determine if the exogenous ORF2 protein expressed from the baculovirus could trans-encapsidate the GFP replicon, infectivity assays were performed with HepG2/C3A cells, the cells most permissive for HEV infection[19]. Cell lysates of transfected and transduced S10-3 cell cultures were prepared in duplicate in 200 μl as described above. Medium was removed from the HepG2/C3A cells and the total cleared cell lysate from each tube was added to an assigned well. Cells were incubated at 37°C for 2.5 hrs with periodic rocking every 15 min. The inoculum was replaced with complete medium and incubation was continued for 6 days. FM observations suggested that the S10-3 cells that received lysates from cells containing both replicon RNA and vBacORF2 expressed GFP in about 5% of naïve HepG2/C3A cells on day 6 post-infection (Figure 5A). On the other hand, lysates from cells receiving replicon alone or vBacORF2 alone, were not able to infect HepG2/C3A cells since at day 6, GFP was not detected. Quantification of GFP-containing HepG2/C3A cells by FACS analysis confirmed the FM results (Figure 5B). Lysates from cells transfected with replicon and later transduced, infected 2% of the HepG2/C3A cells whereas lysates from mock-transfected, replicon-transfected, or vBacORF2-transduced cells did not induce GFP production in HepG2/C3A cells (Figure 5C). The replicon-only lysate control ruled out the possibility that unencapsidated residual transfecting RNA was responsible for GFP production in the HepG2/C3A cells. Therefore, these results clearly demonstrated that exogenously-supplied ORF2 protein is able to trans-complement a replicon deficient in ORF2 protein production and thereby produce intracellular virions that are infectious for cultured cells.

Our results are consistent with the recent report by Xing et al.[14] that HEV virus-like particles formed in insect cells captured some of the template ORF2 RNA used to produce the particles. Whether this capture was fortuitous, specific, or efficient is unclear. In our case, although we were able to infect only 2% of the HepG2/C3A cells, this represented a reasonably efficient packaging of replicon RNA; encapsidation absolutely required co-expression of adequate levels of ORF2 protein and replicon RNA and only 16% of the S10-3 cells contained a functional replicon(Figure 3C) and an estimated 50% contained ORF2 protein. It should be possible to improve the system by further optimizing transfection or transduction parameters but in the meantime our results provide proof-of-principal for trans-encapsidation of HEV genomes by ORF2 and confirm the previous reports that ORF3 protein is not required for generation of infectious virions[18,21]. This trans-encapsidation system should be useful for providing substrates for analysis of neutralizing antibodies or for determining parameters that are necessary for encapsidation of viral RNA, such as packaging signals in the RNA or critical regions or residues in the ORF2 protein.

## Conclusions

This is the first demonstration that the HEV full-length ORF2 protein is efficiently expressed by baculovirus-transduced hepatoma cells. The ORF2 protein trans-complements a replicon that is deficient in capsid protein production and efficiently encapsidates the replicon viral RNA to form stable HEV particles which are infectious for naïve hepatoma cells. This *ex vivo *RNA packaging-system could be further used to study many aspects of HEV molecular biology.

## Competing interests

The authors declare that they have no competing interests.

## Authors' contributions

MKP carried out the molecular studies, and prepared the manuscript. SUE and RHP participated in the design and coordination of the study and prepared the manuscript. All authors read and approved the final manuscript.
